# Effect of a 24-hour praziquantel bath on the haematological and biochemical profile and selected parameters of oxidative stress in grass carp (*Ctenopharyngodon idella*)

**DOI:** 10.17221/89/2025-VETMED

**Published:** 2026-04-27

**Authors:** Radka Dobsikova, Petr Marsalek, Josef Velisek, Jana Blahova

**Affiliations:** ^1^Department of Animal Breeding, Animal Nutrition and Biochemistry, Faculty of Veterinary Hygiene and Ecology, University of Veterinary Sciences Brno, Brno, Czech Republic; ^2^Department of Animal Protection and Welfare and Veterinary Public Health, Faculty of Veterinary Hygiene and Ecology, University of Veterinary Sciences Brno, Brno, Czech Republic; ^3^Research Institute of Fish Culture and Hydrobiology, Faculty of Fisheries and Protection of Waters, University of South Bohemia České Budějovice, Zátiší, Vodňany, Czech Republic

**Keywords:** antioxidant enzymes, blood, lipid peroxidation, quinoline antiparasitic

## Abstract

This study assessed the effect of a 24-hour bath with praziquantel (2 and 4 mg/l) on grass carp (*Ctenopharyngodon idella*) by monitoring the haematological parameters, plasma biochemical profile, and oxidative stress indices. Fish were sampled at 24-, 48-, 72-, and 96-hours post-exposure (hpe). The haematological analysis revealed a significant increase (*P* < 0.05) in the white blood cell count immediately after treatment at both concentrations, with no subsequent changes. Within the plasma biochemical profile, a significant decrease (*P < *0.05) was observed only in chloride (24 hpe at 2 mg/l; 72 hpe at both concentrations) and in magnesium (48 hpe at 2 mg/l). The oxidative stress responses indicated that the gill was the most responsive tissue. In the gill, the catalase and glutathione-*S*-transferase activities increased significantly (*P < *0.05) at 48 hpe at 4 mg/l. Lipid peroxidation in the gill decreased at 48 hpe but increased at 72 hpe following exposure to 4 mg/l. In the hepatopancreas, the glutathione peroxidase activity significantly decreased at 48 hpe at 2 mg/l. In the plasma, significant changes (*P < *0.05) were detected only in the ceruloplasmin activity, which decreased at 72 hpe in the 2 mg/l group. These findings provide essential baseline data supporting the potential future application of praziquantel in aquaculture.

Parasites are among the main limiting factors of the aquaculture industry, as they cause financial losses that account for about 20% of the total production value. The treatment of fish is necessary to prevent direct and indirect economic losses; otherwise, fish may contain infectious larval cestodes or trematodes, which can be finally deleterious to humans. The phylum *Platyhelminthes* comprises a diverse group of parasitic organisms that infect all vertebrates. In captive fish, platyhelminth parasites often cause decreased weight gain and increased mortality (Bader et al. 2019; Kogiannou et al. 2023).

Monogeneans reproduce through a direct life cycle, in which adult helminths live on the definitive host, reproduce sexually, and excrete eggs or larvae that enter the environment and finally infest a new definitive host. On the other hand, cestodes and trematodes use an indirect life cycle, in which an intermediate host is required for the larval development and/or asexual reproduction before returning to the definitive host. Fish are common definitive and intermediate hosts for a wide spectrum of platyhelminth parasites (Bader et al. 2019).

Praziquantel (PZQ) is a synthetic pyrazinoisoquinoline anthelmintic, which is effective against a broad spectrum of cestodes (tapeworms) and trematodes (flukes) and is a mainstay of anti- platyhelminth parasite therapy in both human and veterinary medicine (Bader et al. 2019; Norbury et al. 2022). PZQ’s mechanism of action in a target organism involves disruption of the worm’s tegument, which is characterised by vacuolisation and blebbing (Staudt et al. 1992). PZQ also alters membrane permeability and Ca^2+^ homeostasis by linking to voltage-gated Ca^2+^ channels (Meister et al. 2016).

Many studies have proven the antiparasitic effects of PZQ in fish, e.g., against the infection of barbel by *Diplostomum spathaceum* metacercariae (Zuskova et al. 2018), the infection of common carp by *Atractolytocestus huronensis* (Sudova et al. 2010), the infection of amberjacks, bluefin tuna, and tiger puffer by blood flukes of *Paradeontacylix* spp., *Cardicola* spp., and *Psettarium* spp. (Shirakashi and Ogawa 2016).

PZQ has so far lacked registration for use in aquaculture in Europe. Its use was thus possible only in a veterinary cascade system, regulated by Regulation (EU) 2019/6. Another piece of legislation addressing the use of pharmacologically active substances in animal therapy is Commission Regulation No. 37/2010, which sets maximum residual limits for drugs in foodstuffs of animal origin. In view of the above, a standard withdrawal period of 500 degree-days is strictly determined for fish. Recently, the maximum residual limit (MRL) of PZQ and its isomers in the muscle and skin in natural proportions in fin fish has been set at 20 μg/kg in the Commission Implementing Regulation (EU) 2023/981, which states that the EMA Committee for Veterinary Medicinal Products has adopted an opinion recommending the inclusion of PZQ (the sum of PZQ isomers) in fin fish into the list of allowed substances within the Annex I to Commission Regulation (EU) No. 37/2010.

Therapeutic bath concentrations of PZQ range from 0.25 mg/l to 50 mg/l, depending on bath duration and parasite species. A bath treatment usually involves concentrations up to 10 mg/l of PZQ for prolonged exposure; however, a dip bath typically uses short-time exposure of fish to tens of mg/l of PZQ (Bader et al. 2019).

The goal of the experiment was to investigate the effect of a 24-hour bath exposure of grass carp (*Ctenopharyngodon idella*) individuals at two PZQ concentrations (2 and 4 mg/l), which reflect the relevant dosages used in veterinary practice.

During the test, regular daily sampling was performed to evaluate acute changes in haematological and biochemical parameters, as well as oxidative stress parameters, in the exposed fish. The testing was intended to gain broader knowledge of the anthelmintic’s effect on carp.

## MATERIAL AND METHODS

### Experimental protocol and sample collection

Grass carp (*Ctenopharyngodon idella*) individuals with a mean total length of 24.27 ± 3.47 cm and a mean weight of 181.97 ± 51.34 g were exposed to a 24-hour bath of two concentrations of the anthelmintic praziquantel (2-cyclohexylcarb-oxo-1,2,3,6,7,11b-hexahydro-4H-pyrazino[2,1,-a] isoquinoline) in a powder form (Ecological Laboratories Inc., New York, USA). Due to the low solubility of praziquantel in water, a stock solution was prepared by dissolving the required amount of praziquantel in 96% ethanol (final ethanol concentration: 0.5 ml/l). The fish were obtained from Lnare Fishery, Ltd. (Lnare, Czech Republic) and acclimatised for ten days before the experiment, during which they were fed daily with an appropriate commercial complete pelleted diet for carp. A total of 96 fish individuals were randomly divided and kept in six 100-litre aquaria (i.e., 16 individuals in each aquarium). In the experiment, one control and two experimental groups (with praziquantel at concentrations of 2 and 4 mg/l) were used in duplicate. The experiment was performed in dechlorinated tap water with the following physical-chemical parameters monitored daily in all the aquaria: temperature (20.5 ± 0.6 °C), dissolved oxygen (>94%), pH (7.8 ± 0.3), acid neutralisation capacity (ANC_4.5_, 1.02 mmol/l), chemical oxygen demand (COD_Mn_, 5.11 mg/l), total ammonia (0.02 mg/l), NO_3_^–^ (1.6 mg/l), PO_4_^3–^ (<0.02 mg/l) and a sum of Ca^2+^ and Mg^2+^ 0.95 mg/l.

Biological samples (i.e., blood collected via cardiac puncture into heparinised tubes and selected tissues) for the haematological and biochemical analysis and evaluation of oxidative stress status were obtained from four fish from each aquarium (i.e., a total of eight fish per experimental group) after the 24-hour bath (i.e., 24 h post exposure – hpe) and subsequently at 2-, 3-, and 4-days post-bath (i.e., 48, 72, and 96 hpe, respectively).

The experimental laboratory procedures were conducted in compliance with Czech Regulations No. 166/1996 Coll. and No. 246/1992 Coll. and approved by the Departmental Expert Committee for Authorisation of Experimental Projects of the Ministry of Education, Youth, and Sports of the Czech Republic (MSMT Permit No. MSMT-3126/2021-3).

### Haematological and biochemical indices

For the haematological analysis, heparinised whole blood was used, and all the samples were processed within 3 h after collection. The following indices were determined: red blood cell count, total haemoglobin, haematocrit, and white blood cell count. Subsequently, Wintrobe’s indices were calculated, including the mean corpuscular haemoglobin (MCH), mean corpuscular volume (MCV), and mean corpuscular haemoglobin concentration (MCHC). A detailed description of the analytical procedures is provided in Svobodova et al. (2012). For the biochemical analysis, plasma was obtained from heparinised blood by centrifugation (10 min, 4 °C, 800 × *g*) and stored at –80 °C until examination, which was conducted within one month of collection. The biochemical analyses were performed using a Konelab 20i biochemical analyser (ThermoFisher Scientific, Waltham, USA) and commercial diagnostic kits (Biovendor, Brno, Czech Republic). The following parameters were evaluated: indicators of carbohydrate metabolism (glucose, lactate), lipid metabolism (triacylglycerols, cholesterol), nitrogen metabolism (total protein, albumin, ammonia, creatinine), mineral profile (calcium, phosphorus, chlorides, magnesium), and enzyme activities (alanine aminotransferase – ALT, aspartate aminotransferase – AST, alkaline phosphatase – ALP, creatine kinase – CK, and lactate dehydrogenase – LDH).

### Oxidative stress indices

Oxidative stress markers were assessed in plasma and in tissues from the liver, gill, and caudal kidney. In the plasma, the ceruloplasmin activity and the ferric reducing ability of plasma (FRAP) were determined. In the tissues, the activities of the major antioxidant and detoxifying enzymes were analysed, specifically catalase (CAT), glutathione peroxidase (GPx), and glutathione-*S*-transferase (GST). Additionally, lipid peroxidation was quantified by measuring the thiobarbituric acid-reactive substances (TBARS). Before analysis, tissue samples were homogenised in a cold phosphate buffer (pH 7.2) containing ethylenediaminetetraacetic acid (EDTA) as an antioxidant and subsequently processed under standardised conditions to minimise the enzymatic degradation and prevent secondary lipid peroxidation. All the biochemical parameters were determined spectrophotometrically following established protocols. The enzyme activities were normalised to the protein content of the samples, which was measured using the bicinchoninic acid assay. Protein quantification was performed in parallel to ensure comparability between the tissues and treatment groups. A detailed description of all analytical procedures, including the buffer composition, incubation conditions, and calculations of enzyme activities and metabolite concentrations, is provided in Haluzova et al. (2010) and Mikula et al. (2024).

### Data analysis

Statistical analyses were performed using Unistat v6.5 for Excel (Unistat Ltd., London, UK). The data distribution was first assessed with the Shapiro–Wilk test for normality and Levene’s test for homogeneity of variances. When assumptions of normality and homogeneity were satisfied, a one-way analysis of variance (ANOVA) followed by Tukey’s HSD post hoc test was applied. In cases where the assumption of normality was not met, the Kruskal–Wallis ANOVA followed by Dunn’s post hoc test was used as a non-parametric alternative. Statistical comparisons between the control and exposed groups were always conducted within the same sampling time point. Differences were considered statistically significant at *P < *0.05.

## RESULTS

### Haematological indices

As shown in Table 1, a significant change (*P < *0.05) among the evaluated haematological indices was observed only in the white blood cell (WBC) count at 24 hpe. In both tested concentrations, the WBC values were elevated compared to the control group. At later sampling times, no significant differences were detected (*P *> 0.05). Similarly, no significant alterations (*P *> 0.05) were observed in the red blood cell profile at any sampling time (data not shown).

**Table 1:** Significant changes in the haematological and biochemical indices following a 24-hour praziquantel bath in grass carp (*Ctenopharyngodon idella*)

Indices	Hours post-exposure	Control	2 mg/l	4 mg/l
White blood cell count (G/l)	24 h	22.79 ± 6.45 (23.50)^a^	**31.92 ± 4.33 (31.25)^b^↑**	**30.71 ± 5.57 (29.50)^b^↑**
	48 h	56.21 ± 15.51 (54.00)^a^	51.00 ± 16.17 (52.50)^a^	66.57 ± 13.95 (70.50)^a^
	72 h	76.29 ± 13.98 (82.50)^a^	50.43 ± 11.78 (49.00)^a^	64.64 ± 11.83 (66.50)^a^
	96 h	60.29 ± 6.51 (57.50)^a^	62.86 ± 15.55 (64.50)^a^	75.57 ± 14.24 (79.00)^a^
				
Chlorides (mmol/l)	24 h	119.01 ± 2.52 (119.85)^a^	**115.23 ± 2.55 (115.96)^b^↓**	117.01 ± 2.18 (117.01)^ab^
	48 h	116.40 ± 3.18 (116.16)^a^	118.30 ± 2.01 (118.52)^a^	118.49 ± 2.60 (117.41)^a^
	72 h	119.82 ± 1.25 (119.88)^a^	**113.36 ± 4.15 (115.21)^b^↓**	**113.62 ± 2.41 (114.34)^b^↓**
	96 h	117.24 ± 7.72 (118.12)^a^	114.16 ± 3.52 (112.77)^a^	113.77 ± 3.00 (114.50)^a^
				
Magnesium (mmol/l)	24 h	1.07 ± 0.02 (1.03)^a^	1.10 ± 0.02 (1.10)^a^	1.13 ± 0.01 (1.14)^a^
	48 h	1.05 ± 0.02 (1.06)^a^	**1.13 ± 0.02 (1.13)^b^↑**	1.08 ± 0.02 (1.09)^ab^
	72 h	1.05 ± 0.02 (1.05)^a^	1.05 ± 0.04 (1.06)^a^	1.04 ± 0.01 (1.05)^a^
	96 h	1.05 ± 0.02 (1.06)^a^	1.03 ± 0.02 (1.04)^a^	1.04 ± 0.03 (1.02)^a^

Data are presented as the mean ± standard error of the mean, with the median shown in brackets

^a,b^Significant differences between the groups at the same time point are indicated by different letters (*P* < 0.05); Significant differences compared with the control are highlighted in bold, with arrows indicating the direction of change

### Plasma biochemical indices

The plasma biochemical analysis, including indicators of carbohydrate, lipid, and nitrogen metabolism, selected mineral concentrations, and enzyme activities, revealed significant changes only in the mineral parameters (Table 1). Specifically, the chloride concentrations were significantly decreased at 24 hpe in the 2 mg/l group and at 72 hpe in both tested concentrations compared to the control (*P < *0.05). Magnesium showed a significant increase only in the 2 mg/l group at 48 hpe (*P < *0.05). No significant alterations were observed in the indicators of carbohydrate, lipid, or nitrogen metabolism, nor in the activities of selected enzymes at any sampling time (*P *> 0.05) (data not shown).

### Oxidative stress indices

**Figure 1:**
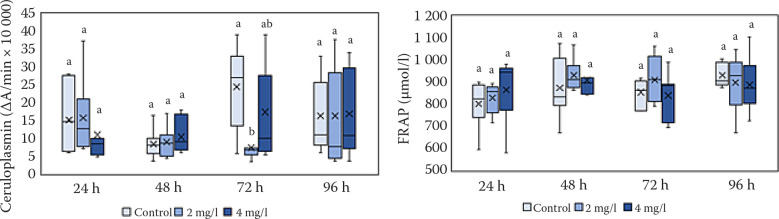
Effect of a 24-hour praziquantel bath on the ceruloplasmin activity and ferric reducing ability of the plasma in grass carp (*Ctenopharyngodon idella*) ^a,b^Significant differences (*P* < 0.05) among the groups are indicated by different superscript letters; In each boxplot, the horizontal line represents the median, the cross indicates the mean, the box extends from the 25^th^ to the 75^th^ percentile, and the whiskers denote the lowest and highest values within 1.5 times the interquartile range

The oxidative stress indices were assessed in the blood plasma (FRAP, ceruloplasmin; Figure 1) and in the tissues of the hepatopancreas, caudal kidney, and gill (CAT, GPx, GST, and TBARS). The results of the analysis of oxidative stress indicators in the tissues are presented in Table 2; for clarity, only those that show significant differences among the groups are shown. In the plasma, significant changes (*P < *0.05) were detected only in ceruloplasmin activity, which decreased at 72 hpe in the 2 mg/l group. In the tissues, the most pronounced alterations were observed in the gill. In the gill, the CAT and GST activities increased significantly in the 4 mg/l group at 48 hpe (*P < *0.05), and, in addition, the GST activity decreased in the 2 mg/l group at 96 hpe (*P < *0.05). In the gill, moreover, the lipid peroxidation was significantly reduced at 48 hpe, but elevated at 72 hpe compared to the control (*P < *0.05). In the hepatopancreas, only GPx and GST activities showed significant changes: GPx decreased at 48 hpe in the 2 mg/l group and at 96 hpe in the 4 mg/l group (*P < *0.05). No significant differences were observed in the caudal kidney at any sampling time (*P *> 0.05) (data not shown).

**Table 2:** Significant changes in the oxidative stress indices following a 24-hour praziquantel bath in grass carp (*Ctenopharyngodon idella*)

Indices	Hours post-exposure	Control	2 mg/l	4 mg/l
**Hepatopancreas**				
GPx (nmol/min/mg protein)	24 h	1 019.7 ± 103.1 (1 176.4)^a^	1 137.7 ± 65.8 (1 152.4)^a^	1 192.8 ± 79.3 (1 206.5)^a^
	48 h	1 247.5 ± 70.4 (1 245.8)^a^	**964.9 ± 67.2 (938.9)^b^↓**	1 031.8 ± 61.7 (1 004.8)^ab^
	72 h	1 014.0 ± 75.7 (990.5)^a^	1 091.7 ± 74.2 (993.3)^a^	1 130.6 ± 70.6 (1 064.6)^a^
	96 h	1 142.1 ± 70.3 (1 126.9)^a^	1 262.3 ± 95.3 (1 322.6)^a^	1 003.2 ± 116.3 (1 038.6)^a^
				
GST (nmol/min/mg protein)	24 h	191.0 ± 9.7 (180.1)^a^	197.5 ± 10.9 (204.2)^a^	191.5 ± 14.5 (190.4)^a^
	48 h	194.4 ± 9.1 (191.1)^a^	170.8 ± 8.4 (173.0)^a^	180.6 ± 10.4 (175.9)^a^
	72 h	182.0 ± 8.3 (175.0)^a^	165.5 ± 20.6 (171.0)^a^	217.3 ± 14.7 (220.8)^a^
	96 h	207.1 ± 8.5 (194.8)^ab^	221.1 ± 9.0 (216.7)^a^	**186.8 ± 9.9 (185.6)^b^↓**
**Gill**				
CAT (μmol/min/mg protein)	24 h	10.5 ± 0.5 (10.4)^a^	14.0 ± 2.2 (14.1)^a^	9.3 ± 1.1 (8.9)^a^
	48 h	7.8 ± 1.0 (6.8)^b^	7.6 ± 0.7 (7.6)^b^	**12.1 ± 0.7 (12.3)^a^↑**
	72 h	11.4 ± 0.6 (11.7)^a^	9.1 ± 1.3 (9.1)^a^	9.1 ± 0.7 (8.7)^a^
	96 h	8.7 ± 0.8 (8.7)^a^	8.9 ± 1.1 (9.0)^a^	11.3 ± 0.9 (10.9)^a^
				
GST (nmol/min/mg protein)	24 h	106.1 ± 16.4 (104.5)^a^	121.4 ± 29.2 (113.0)^a^	117.1 ± 21.1 (118.3)^a^
	48 h	99.0 ± 8.1 (102.8)^a^	98.7 ± 7.2 (97.4)^a^	**127.6 ± 8.0 (126.9)^b^↑**
	72 h	116.2 ± 7.8 (115.2)^a^	138.5 ± 17.3 (147.1)^a^	137.2 ± 7.0 (129.6)^a^
	96 h	104.2 ± 7.2 (106.5)^ab^	**89.1 ± 5.0 (94.0)^b^↓**	117.1 ± 9.7 (109.4)^a^
				
TBARS (nmol/g tissue)	24 h	127.3 ± 12.2 (129.0)^a^	146.2 ± 16.7 (142.5)^a^	153.7 ± 13.2 (163.4)^a^
	48 h	188.4 ± 17.3 (193.9)^a^	160.7 ± 15.2 (162.0)^ab^	**135.3 ± 10.0 (136.6)^b^↓**
	72 h	101.3 ± 13.3 (107.9)^a^	142.0 ± 14.7 (132.0)^ab^	**164.3 ± 11.6 (163.1)^b^↑**
	96 h	198.6 ± 16.0 (203.8)^a^	203.4 ± 10.2 (208.4)^a^	202.4 ± 18.5 (200.0)^a^

Data are presented as the mean ± standard error of the mean, with the median shown in brackets

^a,b^Significant differences between the groups at the same time point are indicated by different letters (*P* < 0.05); Significant differences compared with the control are highlighted in bold, with arrows indicating the direction of change

CAT = catalase; GPx = glutathione peroxidase; GST = glutathione-*S*-transferase; TBARS = thiobarbituric acid-reactive substances assay for estimating the lipoperoxidation

## DISCUSSION

Parasitic infections remain a major challenge in freshwater aquaculture, significantly affecting fish health, growth performance, and overall productivity (Shinn et al. 2015; Buchmann 2022; Velisek et al. 2025). Grass carp (*C. idella*), a widely cultured species in many regions, is particularly susceptible to a range of parasitic pathogens (Zuskova and Velisek 2024). PZQ is a broad-spectrum anthelmintic commonly used to control parasitic infections in fish due to its high efficacy and relatively low toxicity (Bader et al. 2019; Norbury et al. 2022; Zuskova et al. 2022; Dobsikova et al. 2024). Although oral and bath treatments with PZQ have been widely used in aquaculture and their antiparasitic efficacy has been well documented (Mitchell 2004; Mitchell and Darwish 2009; Zuskova et al. 2018; Zuskova and Velisek 2024), there is still a limited number of studies addressing the potential physiological consequences of these treatment methods in grass carp. In particular, the effects of a short bath administration on the haematological, biochemical, and oxidative stress parameters remain insufficiently explored. Therefore, our study provides valuable insights into the broader physiological impacts of a 24-hour bath treatment with PZQ in grass carp (*C. idella*). We primarily focused on low PZQ concentrations, corresponding to the relevant dosages used in fish therapy. The observed alterations in the haematological, biochemical, and oxidative stress parameters indicate that, although PZQ is generally well tolerated, it can elicit measurable biological responses in a time- and dose-dependent manner.

Haematological parameters are considered sensitive indicators of the fish physiological condition and their responses to various stressors or toxicants. Changes in the red and white blood profiles can reflect both the direct effects of the tested compound and the secondary physiological responses to various environmental stressors (Witeska and Kondera 2022; Witeska et al. 2023). In the present study, the short-term (24 h) bath exposure of grass carp (*C. idella*) to PZQ resulted in a significant alteration (*P < *0.05) only in the total leukocyte count, which increased at both tested concentrations (i.e., 2 and 4 mg/l). The elevation in the white blood cell count was observed exclusively at 24 hpe, whereas no significant differences were detected at later sampling intervals. The other haematological indices, including the erythrocyte indices, remained unaffected throughout the experimental period. The transient leukocytosis observed at 24 hpe likely reflects an acute stress or immune response to the treatment (Davis et al. 2008). Similar short-term increases in the white blood cell count have also been reported in fish following exposure to various pollutants, parasitic infections, or treatment with antiparasitic compounds (Pereira et al. 2012; Jeronimo et al. 2014; Saravana et al. 2017; Kumar 2023). Although PZQ primarily acts by disrupting the tegument of parasites, it has also been shown to exert immunomodulatory effects in fish (Polinski et al. 2014; Soltanian et al. 2017). Such immune modulation may be associated with the activation of the hypothalamus–pituitary–interrenal axis, leading to increased cortisol secretion and subsequent changes in the leukocyte distribution. Elevated leukocyte counts shortly after the exposure may therefore indicate the mobilisation of defence mechanisms rather than a toxic effect *per se*. The return of leukocyte levels to baseline within 48–96 hpe suggests the rapid physiological recovery and good tolerance to PZQ in grass carp (*C. idella*). This pattern is in line with previous observations in other fish species, in which acute exposure to PZQ induced only transient changes in the selected haematological or biochemical parameters, without causing lasting effects (El-Banna et al. 2008; Sudova et al. 2009). The absence of changes in erythrocyte indices further supports the notion that PZQ does not compromise the oxygen transport capacity or induce haemolytic stress under the tested conditions. Altogether, these results indicate that PZQ, when used at therapeutically relevant concentrations, induces mild and reversible haematological responses in grass carp (*C. idella*), likely associated with transient stress and immune activation rather than systemic toxicity.

Plasma biochemical parameters, among others, provide important insights into the physiological and metabolic status of fish and are commonly used to evaluate sublethal effects of various environmental stressors (Blahova et al. 2025; Bojarski et al. 2025). In the present study, a wide range of indicators reflecting the carbohydrate, lipid, and protein metabolism, as well as the mineral balance and enzymatic activity, were analysed. Overall, most plasma biochemical parameters remained stable across the experimental period, suggesting that PZQ at the tested concentrations exerts minimal systemic metabolic disturbance. However, specific alterations were observed in the mineral parameters, such as the magnesium and chloride levels. Magnesium was elevated at 48 hpe in the 2 mg/l group, while chloride decreased at 24 hpe in the 2 mg/l group and at 72 hpe in both tested concentrations. Importantly, these changes did not show a clear concentration-dependent pattern and, similar to the haematological responses, were transient, returning to baseline values by 96 hpe. Similar short-lived alterations were reported in Nile tilapia (*Oreochromis niloticus*) by El-Banna et al. (2008), in which a PZQ-supplemented feed induced temporary changes in the plasma glucose, total protein, and globulin concentrations at 48 hpe, with the values returning to normal after 96 hpe. Furthermore, Soltanian et al. (2017) observed that a 24-hour PZQ bath in common carp (*Cyprinus carpio*) at concentrations of 0.5, 1.5, and 5 mg/l had no effect on the plasma glucose, but induced a delayed increase in the total protein at two higher concentrations (i.e., 1.5 and 5 mg/l) that became significant only 21- and 28-days post-exposure. A comparable study by Sudova et al. (2009) examined the effects of a single oral PZQ administration in common carp (*C. carpio*) at concentrations of 30 and 50 mg/kg and reported a transient decrease in the plasma total protein (only 30 mg/kg), globulin, and glucose concentrations after 24 hpe, which normalised by 96 hpe. The only exception was the ALT activity, which showed a significant increase at 96 hpe in both concentrations – almost doubling compared to the control values – suggesting slight hepatocellular damage potentially associated with PZQ metabolism. Taken together, these findings indicate that PZQ generally produces reversible biochemical alterations in fish, with the magnitude and duration of effects depending on the species, dose, and route of administration. The changes observed in our study, therefore, represent short-term, non-toxic physiological adjustments rather than a persistent metabolic disruption.

Oxidative stress serves as a sensitive biomarker of cellular disturbance and is widely used to assess the sublethal toxic effects in fish. It reflects the dynamic balance between the generation of reactive oxygen and nitrogen species and the activity of antioxidant defence systems that safeguard cells from oxidative damage (Lushchak 2014; Formicki et al. 2025). In the present study, the assessment of oxidative stress markers showed that PZQ induced only mild and short-lived effects. The antioxidant enzyme activities and lipid peroxidation analysed in the selected tissues and plasma indices such as FRAP and ceruloplasmin provided complementary information on the systemic redox balance. Significant responses were observed primarily in the gill tissues, which are directly exposed to the external environment and thus represent the first site of contact with waterborne substances. In fish exposed to the lower PZQ concentration, a significant decrease in the GST activity was found at 96 hpe. In the fish exposed to the higher PZQ concentration, both the GST and CAT activities increased significantly at 48 hpe, accompanied by a temporary decrease and subsequent elevation in the lipid peroxidation in the gill. In the hepatopancreas, only a short-term reduction in the GPx (in 2 mg/l at 48 hpe) and GST (in 4 mg/l at 96 hpe) activities was recorded, and no significant alterations were observed in the caudal kidney. The plasma ceruloplasmin activity also showed a brief decline at 72 hpe in the lower concentration group. Overall, these results indicate that PZQ can induce limited oxidative stress, mainly in the tissues directly involved in environmental interactions and detoxification, such as the gill and hepatopancreas. However, the lack of consistent, concentration-dependent trends suggests that these effects are reversible and adaptive rather than indicative of sustained oxidative stress or systemic toxicity, similar to the other haematological and biochemical indices discussed earlier. Comparable results were reported by Velisek et al. (2025), who found a significant elevation of the GST activity in the whole-body homogenates of grass carp (*C. idella*) exposed to 2 mg/l PZQ after 29 days with the embryo-larval toxicity test, indicating the effective activation of detoxification pathways that maintained redox homeostasis. No changes were detected in the other antioxidant enzymes or lipid peroxidation. In contrast, an embryo-larval toxicity test with common carp (*C. carpio*) proved reduced catalase (CAT) and superoxide dismutase (SOD) activities after long-term exposure (35 days) to 3 and 4 mg/l of PZQ, suggesting that the sensitivity to oxidative disturbance may vary depending on the species and developmental stage (Velisek et al. 2022). Furthermore, Zuskova et al. (2018) studied barbels (*Barbus barbus*) naturally infected with *Diplostomum spathaceum* and *Pomphorhynchus laevis*, in which therapeutic PZQ baths (10 and 20 mg/l) significantly altered the activities of antioxidant enzymes (CAT, SOD, GR, and GST) and glutathione concentration in the liver and muscles. Since the fish were infected, these changes likely reflected a combined effect of the antiparasitic treatment and parasite-induced oxidative stress, making a direct comparison with uninfected fish less straightforward.

Our study demonstrates that a 24-hour PZQ bath at concentrations of 2 and 4 mg/l induces moderate physiological changes in grass carp, particularly affecting haematological parameters, plasma electrolyte balance, and oxidative stress responses in hepatopancreas and gill tissue. Importantly, these effects were short-lived. This indicates that PZQ at therapeutically relevant concentrations exerts only transient, reversible physiological effects rather than persistent or cumulative toxicity. While no severe adverse effects were observed, the detected sublethal alterations underscore the importance of careful dose selection and ongoing monitoring when using PZQ in aquaculture. These findings contribute to a better understanding of PZQ’s safety profile and its potential role in disease management, and they highlight the need for further research into the long-term effects and species-specific responses. For a comprehensive assessment of the potentially undesirable effects of PZQ on grass carp, it would be appropriate to focus further research on evaluating the effects of a wider range of tested concentrations, which would provide an overall view of the physiology of the internal environment of the exposed individuals.
